# Models of care for non-communicable diseases for displaced populations in Iraq: a scoping review

**DOI:** 10.1186/s13031-022-00474-w

**Published:** 2022-07-15

**Authors:** Benjamin Schmid, Éimhín Ansbro, Emmanuel Raju, Ruth Willis, Nazar Shabila, Pablo Perel

**Affiliations:** 1 Centre for Global Chronic Conditions, London School of Hygiene and Tropical Medicine, London, UK; 2 Department of Community Medicine, College of Medicine, Hawler Medical University, Erbil, Iraq; 3 Department of Non-Communicable Disease Epidemiology, Faculty of Epidemiology and Population Health, London School of Hygiene and Tropical Medicine, Keppel Street, London, WC1E 7HT UK; 4 Global Health Section and Copenhagen Centre for Disaster Research, Department of Public Health, University of Copenhagen, Copenhagen, Denmark; 5 Research Fellow in Social Science, Department of Health Services Research and Policy, Faculty of Public Health and Policy, London School of Hygiene & Tropical Medicine, London, UK; 6 Unit for Environmental Sciences and Management, African Centre for Disaster Studies, North-West University, Potchefstroom, South Africa

**Keywords:** Noncommunicable disease, Conflict, Displaced populations, Complex emergencies, Programmes, Implementation

## Abstract

Non-communicable diseases (NCDs) are the leading cause of death and disability globally. Their importance in humanitarian settings is increasingly recognised, but evidence about how best to address NCDs in these setting is limited. This scoping review aimed to explore models of NCD care for displaced populations in Iraq, in order to build evidence to design context adapted models of care. A search of key databases (Medline, Embase, Scopus, EconLit, Global Health, Web of Science, and the Iraqi Academic Scientific Journals) was conducted and complemented with grey literature and snowballing searches. Documents were included if they referred to models of NCD care for displaced populations. We synthesised the data using a conceptual model of care framework. The findings were reported according to the PRISMA guidelines for scoping reviews. We identified 4036 documents of which 22 were eligible for inclusion. Only six documents were peer-reviewed studies with most being internal reports, commentaries, or press releases. Of the 14 documents that reported on their methods, most applied quantitative approaches (n = 7), followed by mixed-methods (n = 5) and qualitative approaches (n = 2). Only one document reported on outcome data and none applied longitudinal study designs. Documents generally described individual framework dimensions, mostly centring around medicines, facility-based services, and selected access dimensions. Most dimensions had few or no references. The most common model for displaced populations in Iraq was primary-level centred care that complemented or supported existing—mostly tertiary—public health system structures. Additionally, private facilities played an important role and were frequently accessed by displaced populations in most settings. Quality of care, particularly patient-perceived quality, emerged as a critical factor for designing context-adapted models of NCD care. This review also identified a strong regionality of NCD care, particularly in terms of access rates and barriers. We concluded that there is a scarcity of evidence on the effectiveness of models of NCD care for displaced populations in Iraq, calling for capacity building initiatives focused on implementation research and evaluation.

## Background

Over the last three decades there has been a major shift in the duration, frequency, and number of people affected by humanitarian crises, fuelled by disasters, armed conflicts or both [1–4]. Simultaneously, the characteristics of the populations affected by humanitarian crises and their needs are changing, including non-communicable diseases (NCDs) becoming increasingly prevalent [5]. This transition is driven by an advancing epidemiological transition in low-income countries and by middle-income settings being affected by humanitarian crises, particularly since the Balkan and Caucasus wars in the 1990s [5].

Humanitarian actors are increasingly acknowledging the need to address the chronic care needs of people living with NCDs in their programming, which historically evolved to provide acute, episodic care [5–8]. Major gaps in the NCD care provision by humanitarian actors were highlighted in the last decade [9, 10]. Progress has been achieved on many of these including the integration of NCDs into the Inter-Agency Emergency Health Kit and the publication of multiple important reviews around NCDs in humanitarian settings [7, 11–15]. Despite the increasing attention and research, uncertainties of (cost-) effective models of care remain due to limited evidence of impact and quality of research publications [7].

This review contributes to a research programme—anchored within the “Partnering for Change” partnership—aiming to address the knowledge gap around cost-effective models of care for NCDs in humanitarian settings globally, with case studies in Iraq and Lebanon. This review’s focus is limited to Iraq. Iraq is an upper-middle-income country with substantial internal displacement, a population affected by protracted and recurring conflict and an advanced epidemiological transition [16–19]. NCDs accounted for 65% of the burden of disease in 2019 and have been included in its national care package since 2009 [20, 21]. Despite the national public health system’s resilience and historic strength, decades of armed conflicts, economic crises, and weak governance have severely impacted its capacity [17, 22, 23]. Care delivery is situated in a complex context of power relationships with governments in central Iraq and in the northern semi-autonomous Kurdistan Region of Iraq (KRI). Next to national actors, the healthcare system is made up of prominent international development and humanitarian actors and a strong, but largely unregulated, private sector [23].

## Methods

**Fig. 1:**
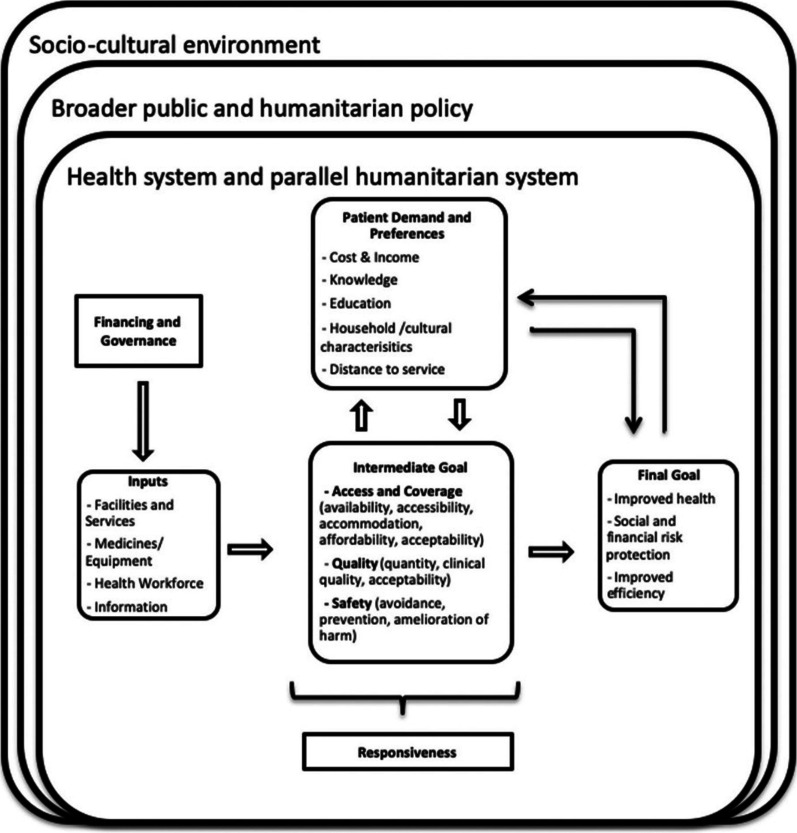
Conceptual framework for a model of care for NCDs in humanitarian crises [7]

This scoping review aims to explore models of care for NCDs for displaced populations in Iraq. This review was guided by a model of care conceptual framework that was developed for the overarching research programme to improve comparability across settings (see Fig. 1).

We used scoping review methodology to map existing evidence and identify research gaps, applying the adapted PRISMA standards (Preferred Reporting Items for Systematic Reviews and Meta-Analyses) for scoping reviews [24, 25]. This method was appropriate considering the review’s broad research aim [26].

### Model of care framework

The model of care framework (see Fig. 1) was developed for the overarching study to assist with data analysis and reporting of heterogeneous models across diverse settings. We considered a model of care as the “*overarching design for the provision of a particular type of health care service*” [27].

The framework was based on an adapted version of the World Health Organization (WHO) health building blocks framework and existing models of care [28]. These included the components of high-quality health systems, a model of NCD care framework for low- and middle-income countries (LMICs), economic models and social system approaches to describing a healthcare system, and access to care concepts [29–34]. Adaptations intended to address earlier criticisms of overly mechanistic blocks and to better capture ‘patient-centredness’. A description of the specific conceptual framework dimensions was provided in the original publication [7]. This model of care framework was used to inform and guide data extraction and analysis.

### Data collection process

The PICO(S) tool (see Table 1) depicts the review’s eligibility criteria. The disease focus—on diabetes mellitus (DM), hypertension (HTN), and cardiovascular disease (CVD)—was based on local disease burden and global relevance, previous author recommendations, and the feasibility of addressing these diseases jointly and at a primary level [7, 35, 36]. The search focused on displaced populations, both internally displaced populations (IDPs) and refugees, to capture adaptations made to cater for populations affected by humanitarian crises. Keywords included both conflict- and disaster-caused displacement based on an initial review of the EM-DAT disaster database [37]. We included care provided through the country’s public and private formal healthcare systems, and through ‘parallel’ systems provided by humanitarian actors, such as those providing complimentary service and those integrating with existing structures. Documents were included if they described a humanitarian actor’s models of care even if they did not specify a focus on displaced populations. Documents with data about people living with NCDs as a subgroup were only included if the data were stratified or the overall arguments applied to the whole group under study. If the same data were available across multiple resources, the newer source was used and other documents were excluded.

**Table 1:** PICO(S) tool describing the inclusion and exclusion criteria used in the review

	Inclusion criteria	Exclusion criteria
Population of interest	People living with NCDs (defined as CVD, HTN and DM) or CVD risk factors	Studies addressing specific NCDs other than the ones described
	Displaced persons (IDPs or refugees)	Veterans and former or active combatants
	Iraq or Iraqi Kurdistan	People resettled or living in high-income countries (HIC)
Intervention	All health system levels and care not traditionally facility-based by both parallel and public/private actors	Military hospital with HIC resources
	Care delivered across the continuum of care, including prevention, diagnosis, treatment and palliative care	
	Access to care	
Outcome and study types	None, patient outcomes, system indicators	
Study design	Quantitative, qualitative and mixed-methods designs Secondary data if primary source not available	Commentaries, reviews, editorials, opinion pieces and weekly or monthly humanitarian updates
Publication date and language	Publication date between 1990 and 2020	Publication prior to 1990
	English	Not in English

The three-stage data collection process included database, grey literature, and snowballing searches. Seven databases (OVID MEDLINE, OVID EMBASE, Web of Science Core Collection, Scopus, Iraqi Academic Scientific Journal, EconLit bibliographic databases, and Global Health) were systematically searched in December 2020. The search consisted of four different keywords: (1) NCDs and risk factors (e.g. diabetes OR “hypertens*” OR “cardiovascular disease*” OR obesity), (2) model of care dimensions (e.g. “intervention$” OR affordability), (3) population group (e.g. “conflict-affected” OR displaced OR humanitarian) and (4) geographical focus (e.g. Kurdistan OR Iraq*). The snowballing search consisted of scanning the references, citations, and other publications by the first author of records identified in the database search. The grey literature search included key humanitarian actors’ resources and platforms (e.g. International Committee of the Red Cross (ICRC), Médecins Sans Frontières (MSF), United Nations High Commissioner for Refugees (UNHCR), International Rescue Committee (IRC), WHO, ReliefWeb), based on previous research approaches [13, 15]. A full list of platforms and organizational websites that were checked was added in ANNEX A. The search keywords and approach were adapted according to each platform’s search functions. An example search is provided in ANNEX B.

### Study selection and data extraction

**Fig. 2:**
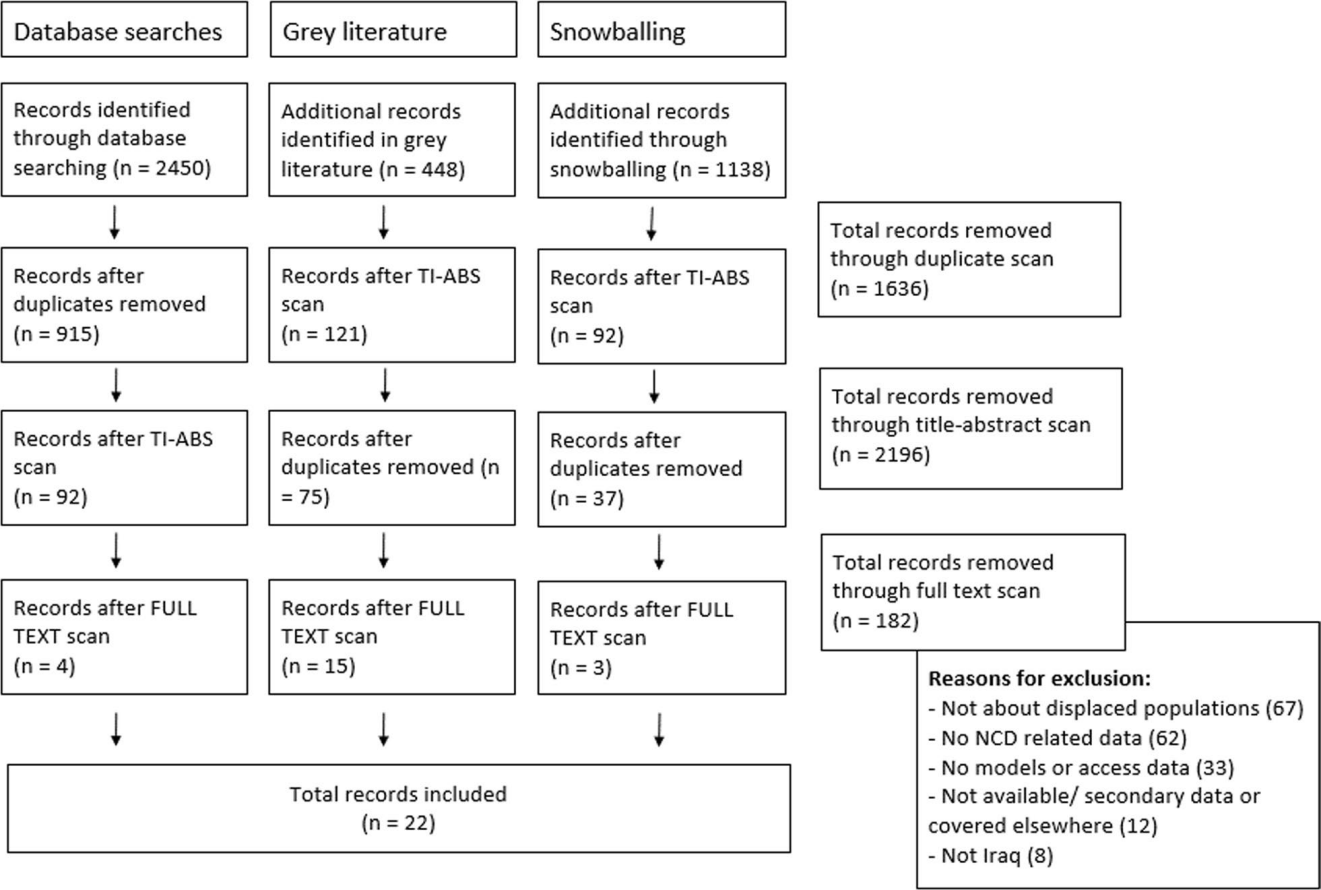
PRISMA search and scan flowchart

The citations from the search results were imported into EndNote X9 desktop [38]. After the removal of duplicates, the document’s title and abstract were scanned for inclusion. The remaining documents were full-text scanned for final inclusion. All scans were guided by the criteria in Table 1. The full-text scan was conducted by at least two authors for each document (BS and RW or NS) and disagreements were moderated by a third author (EA). Exclusion reasons are provided below (see Fig. 2). The data were extracted by the first author for each framework domain and dimension separately, using an Excel-based tool, and the results presented using the same structure. Additional contextual data was plotted, including for example the type of humanitarian setting, study population, research methods or study characteristics. A dimension was considered to be addressed if related data was provided, implications were discussed, recommendations given or challenges described. There was no specific process of obtaining or confirming data from included documents’ authors.

## Results

### Study selection

The literature search yielded 4036 citations in total, of which 22 were eligible for data extraction. The commonest reasons for full-text exclusions were that documents did not focus on or stratify data for displaced populations (n = 67) or the included NCDs (n = 62). A flowchart of the literature search and scan is provided below (see Fig. 2). For the grey literature and snowballing search, title-abstract scans were done prior to duplication removal due to the limitations of the search platforms. The depth of data was minimal and documents were included even with scant references to models of NCD care for displaced populations.

### Study characteristics

Characteristics of the 22 included documents are described below (Table 2) and a summary of each document is provided in Table 3. Most of the included documents were not peer-reviewed and were thus considered grey literature (73%, n = 16). The majority of those were internal reports by humanitarian organizations. Around 20% of the included documents were press releases or commentaries.

**Table 2:** Characteristics and distribution of the included documents

**Study design:** Peer-reviewed publications (n = 6), five of which were descriptive, cross-sectional studies, mostly quantitative [40–42] rather than qualitative [43], mixed-method [60] or a commentary [48]. The remaining were grey literature (n = 16) and comprised internal reports [39, 57, 51, 51–53, 55–58], a commentary [59], a thesis [46], and news or press releases [54, 47, 49, 50]. Of the grey literature documents, eight reports and the thesis provided methodological descriptions. Most used quantitative cross-sectional designs [58, 55, 56, 46]. Mixed methods were used in four reports, using household surveys, school visits, document or secondary data reviews, focus groups, interviews, and direct observations [39, 53, 52, 44]. One report used qualitative interviews and focus group discussions [45]
**Study population:** Refugees (n = 6) [44–46, 45, 59, 46], IDPs (n = 12) [39–43, 48–51, 53, 49, 50], specific organization’s beneficiaries [58], or multiple stakeholders including both IDPs/refugees and officials or healthcare providers (n = 3)
**Study location:** On national level or in multiple governorates (n = 10) [40, 41, 60, 48, 58, 55, 44, 58–60] or individual governorates (n = 12) [39, 42, 43, 57, 51, 49–54, 50], with no one governorate covered by more than two documents. The studies were mostly in the Kurdistan Region of Iraq (n = 12) [40, 41, 44–46, 44, 56, 45, 54–56, 49], rather than in Iraq (n = 6) [39, 42, 43, 48, 57, 53] or in the Disputed territories of Northern Iraq (n = 4) [60, 58, 47, 50]. Studies were mostly camp-based (n = 10) [40, 41, 60, 52, 55, 44, 46, 54, 47, 50], rather than in community-based (n = 4) [39, 56–58], or both settings (n = 6) [42, 43, 51, 45, 59, 49]. In two studies the care setting was not specified [48, 53]
**Care provider:** Public or private and parallel healthcare providers simultaneously (n = 15) [39–41, 43–45, 52, 58, 55, 54–58, 50], solely parallel systems (n = 6) [46–48, 59, 46, 47], or solely public/private systems (n = 1) [42]
**Study period:** Published between 2015 and 2020 (n = 17) [39–44, 46–50, 56, 46, 56–58, 50], between 2010 and 2014 (n = 5) [51, 52, 55, 45, 59] and none prior. Study duration (n = 14) was between 1 and 5 months, except for one 34-month study [43]
**Target NCD condition:** NCDs in general or more than one NCD (n = 19) [39–45, 51, 53, 51–60] or DM (n = 3) [48, 46, 50]

**Table 3:** Detailed descriptions of the 22 included documents

Citation	Title	Setting and population	Design and size
Baxter et al. [43]	Access to care for non-communicable diseases in Mosul, Iraq between 2014 and 2017: a rapid qualitative study	Displaced persons attending MSF clinic in Mosul with DM/HTN over the age of 18. Reports on care by both parallel and public/private care	Qualitative, cross-sectional study based on semi-structured questionnaire-based interviews; sample: 15
Boulle et al. [48]	Challenges associated with providing diabetes care in humanitarian settings	Describes care provided through mobile clinics to serve displaced populations in Iraq. Care by parallel system (MSF)	–
Bruaene et al. [51]	Evaluation of the DG ECHO’s Action In Response to the Iraqi Crisis (2007–2009)	Describes care provided by parallel systems (Directorate-General for European Civil Protection and Humanitarian Aid Operations; DG ECHO) for displaced populations in Iraq with CVD, DM, HTN	–
Cetorelli et al. [40]	Prevalence of non-communicable diseases and access to health care and medications among Yazidis and other minority groups displaced by ISIS into the Kurdistan Region of Iraq	Random sample of IDP households residing in official camps in the Kurdistan region including people living with various NCDs (CVD, DM, HTN, musculoskeletal conditions). Reports on care by both parallel and public/private systems	Quantitative, cross-sectional survey; sample: 1300 households (8360 members)
Cetorelli et al. [41]	Health needs and care seeking behaviours of Yazidis and other minority groups displaced by ISIS into the Kurdistan Region of Iraq	Random sample of camp households in the Kurdistan region including people living with various NCDs (CVD, DM, HTN, musculoskeletal conditions). Reports on care by both parallel and public/private systems	Quantitative, cross-sectional survey; sample: 1300 households (8360 members)
Jadoo et al. [42]	The impact of displacement on the social, economic and health situation on a sample of internally displaced families in Anbar Province, Iraq	Convenience sample of head of households of displaced families in Anbar province. Reports on multiple NCDs (HTN, DM, arthritis, heart diseases, asthma) and care provided by the public/private system	Quantitative, cross-sectional survey; sample: 355 household heads
IRC [53]	Multi-sectoral need assessment. Western Anbar—Qaim and Anah, Iraq	Randomly selected households in Western Anbar as well as education and legal professionals, and community members. Reports on multiple NCDs (mainly DM, HTN) and care provided by the public/private system	Mixed-methods, quant.: household survey and school visits; qual.: focus group discussions, key informant interviews and direct observation form; sample: 60 + 8 + 7 (for quantitative/qualitative components)
Kiani [52]	Rapid Needs Assessment: Situation of people with disabilities in 4 camps in Erbil—Kawergosk, Darashakran, Qushtapa and Basirma	Purposive sample of local and international service providers and people living with disabilities in four refugee camps in the Kurdistan region. Reports on multiple NCDs (CVD, DM, chronic obstructive pulmonary disease, cancer, HTN, others) and care by both parallel and public/private systems	Mixed-methods, document review, surveys (quantitative and qualitative), interviews and observation; sample: 1042 (for quantitative data)
Lafta et al. [60]	Needs of Internally Displaced Women and Children in Baghdad, Karbala, and Kirkuk, Iraq	Cluster random sample of women in IDP families living in informal settlements in Baghdad, Karbala or Kirkuk. Reports on multiple NCDs (CVD, DM, asthma, arthritis) and care by both parallel and public/private systems	Mixed-methods, cross-sectional survey; sample: 1216 families (with 3665 children)
REACH [55]	Multi-Sector Needs Assessment (MSNA) of Syrian Refugees in Camps, Kurdistan Region of Iraq	Random sample households in refugee camps in the Kurdistan region. Reports on multiple NCDs (CVD, DM, HTN, asthma, others) and care by both parallel and public/private systems	Quantitative, cross-sectional survey; sample: 804 households
REACH [44]	Multi-Sector Needs Assessment (MSNA) of Syrian Refugees residing in Camps, Kurdistan Region of Iraq	Random sample of households in refugee camps in the Kurdistan region. Reports on multiple NCDs (CVD, DM, HTN) and care by both parallel and public/private systems	Mixed-methods, cross-sectional survey, field observation, dialogue with camp management and secondary data review; sample: 2678 households (13,390 persons)
REACH [56]	Multi-Sector Needs Assessment of Syrian Refugees Residing in Host Communities: Iraq	Random sample of refugee households in the Kurdistan region. Reports on multiple NCDs (CVD, DM, HTN, asthma, liver/ stomach/ kidney problem, cancer) and care by both parallel and public/private systems	Quantitative, cross-sectional survey; sample: 1734 households
Sa'Da et al. [59]	Humanitarian and medical challenges of assisting new refugees in Lebanon and Iraq	Describes care by parallel systems for refugees in Iraq, reporting on multiple NCDs (CVD, DM, HTN)	–
Shamsi [46]	Prevalence, management and control of diabetes mellitus among Syrian refugees in Duhok governorate, Kurdistan Region of Iraq–a cross sectional study in the camp of Domiz 1	Waiting room study sample of DM patients during a regular check-up at a camp-based Primary Health Care Centre (PHCC) in Duhok. Reports on care by parallel systems	Quantitative, cross-sectional survey; sample: 204
UNHCR [45]	Iraq—Joint Rapid Needs Assessment of Syrians in the Kurdish Region	Describes care for Syrian refugees in the Kurdistan region, through heads of community-based organizations and community leaders. Reports on multiple NCDs (mainly DM, HTN) and care provided by both parallel and public/private systems	Qualitative, participatory assessment: semi-structured interviews in focus group discussions; and key informant interviews; sample: 500 persons
WHO [49]	Mobile clinics bring services to communities in need	Describes care for displaced persons in Iraq. Reports on multiple NCDs (CVD, DM, HTN, skin disease) and care by both parallel and public/private systems	–
ACAPS [57]	Iraq: The return to Sinjar	Describes care for IDPs and returnees to Sinjar district. Reports on NCDs (unspecified) and care by both parallel and public/private systems	–
ICRC [54]	Chronic Diseases: The Forgotten War Trauma	Describes care for IDPs, mostly Yazidis, living with NCDs (DM, HTN) at the Sharia Camp in Duhok. Reports on care by both parallel and public/private systems	–
MSF [47]	Iraq: Imminent Laylan camp closure risks humanitarian consequences	Describes care for IDPs at Laylan camp in Kirkuk. Reports on NCDs (unspecified) and care provided by the parallel system (MSF)	–
Oxfam et al. [58]	COVID-19 – Impact on Older People – Rapid Needs Assessment	Sample of Oxfam beneficiaries aged < 50 years in Anbar, Diyala, Kirkuk, Ninewa, and Salah al-Din. Reports on multiple NCDs (DM, HTN, CVD, others) and care by both parallel and public/private systems	Quantitative, cross-sectional survey; sample: 605 people
CARE [39]	CARE Endline Evaluation Survey: Zummar Sub-District–Ninawa Governorate, July 2020	Purposive and random sampling of host population, returnees and IDPs in Zummar sub-district aged 18–60 years. Reports on NCDs (not specified) and care by both parallel and public/private systems. Provides process and patient-reported outcome data	Mixed-methods, qualitative and quantitative approaches (key informant interviews, household visits, focus group discussion and interviews); sample: 750 (for quantitative; NCD beneficiaries only)
WHO [50]	Internally displaced health workers support services for IDPs in Kirkuk	Describes care for IDPs in six camps in Kirkuk, mostly from Hawija. Reports on DM and care by both parallel and public/private systems	–

Only 14 of the 22 (63%) included documents reported on their methodology, which included five peer-reviewed studies and nine grey literature reports. Of these 14 documents most applied a cross-sectional design using quantitative surveys. Five combined survey data with qualitative interviews or focus groups, and two documents used qualitative methods only. All but one (an internal report that reported on process and patient-reported outcomes) were descriptive [39]. No documents provided clinical outcomes such as blood pressure control, complications, or mortality. Further details on the distribution of the included documents’ characteristics can be found in Table 2.

### Data synthesis

Results are presented according to the conceptual framework domains and dimensions. To consider the importance of contextual factors, results were stratified based on geographical location as well as service provider, comparing camp- to community-based settings, where available [7]. Due to the general scarcity and limited depth of available data, with most documents being internal reports or press releases, we included items with minimal references to models of NCD care.

Most documents described individual framework dimensions. The most frequently addressed dimensions were accessibility (77%, n = 17), **availability** (68%, n = 15), and **facility-based services** (64%, n = 14). Evidence mostly centred around the traditional health system building blocks or selected access dimensions. The contextual dimension of sociocultural environment was addressed by 77% (n = 17) of documents, and that of broader public and humanitarian policy by 14% (n = 3) of documents. All but three conceptual framework dimensions, **financing and governance, safety** and **education**, were addressed by at least one document. A detailed overview of the addressed dimensions is available in ANNEX C.

#### Health system inputs

For the **facility and services** dimension, most documents made reference to camp-based populations. They typically referred to either a joint response by camp-based PHCCs, public hospitals and private clinics or pharmacies or the provision of care by camp-based PHCC services [40–47]. In some settings, mobile clinics were deployed, with the aim of improving access and increasing flexibility [48–50]. Documents did not generally describe the type of services provided, with some exceptions. Amongst those providing some level of detail were a camp-based PHCC in the KRI reportedly delivering both preventive and basic curative services as well as a mobile clinic providing basic follow-up and education as part of a “light” model of care [40, 48]. One document detailed services for non-camp-based populations by a Directorate of Health (DoH)-run PHCC supported by CARE International (Cooperative for Assistance and Relief Everywhere) [39]. Their services included NCD consultations, medicines and laboratory tests. Except for one document recommending tertiary care for NCD patients, all authors described a primary care centred approach [51]. No papers described **community-based services**, despite some authors reiterating their importance, particularly for people living with disabilities [41, 52]. Descriptions of the **health workforce** were limited to three documents, all centred at PHCCs. One reported staffing of the PHCC by a doctor and medical auxiliaries [40], while in another project specialists were present in the afternoon hours [39]. One further document recommended increasing the number of female service providers in camp-based PHCCs [53].

Documents rarely described specific details around **medicines and equipment**, such as treatment approaches or related infrastructure. One grey literature study on DM patients in a camp-based setting—Domiz 1 in Duhok—described specific treatments [46]. They noted that all patients with Type 1 DM were treated with insulin, mostly with Insulin Mix morning and night (64%), and patients with Type 2 DM were treated with Metformin and Glibenclamide bi-therapy (55%) or Metformin monotherapy (43%). Patients were also treated with cardiovascular and anti-hypertensive medicines. An internal project evaluation of a PHCC-based model of care supported by CARE mentioned the inclusion of medicines provision, quality control, and a supply chain needs assessment [39]. Other insights on medicines and equipment were provided by commentaries or press releases. Two documents anecdotally described improved medicines storage and good availability in mobile clinics [48, 49]. A WHO news article also referenced the use of the interagency emergency health kit in 2016 [50].

Under the **information** dimension, no documents detailed the use of health information systems. The only reference to a health information system was from a press release referring to medical files existing at MSF camp-based facilities [47]. Similarly, there were references to ‘basic patient education’ as part of MSF’s mobile clinics model of care, while no specific descriptions of their content or approaches were outlined [48]. One author called for patient education focused on healthy eating and patient responsibility, in response to complications being observed in DM patients on medications [46].

Virtually no papers collected **outcome data** with the only exception being an internal evaluation report of a CARE project with an NCD component, next to maternal health services, at a DoH-run PHCC in Zummar district [39]. The project reported on process and patient-reported outcomes. The project’s NCD model of care was built around the provision of essential medicines and laboratory supplies, the extension of its opening hours and the presence of a specialist during afternoon hours. The process indicators included staff perception of the supply rate and quality of medicines (92% reporting “very good”), the implementation of a supply chain and medicines needs assessment, and increased patient numbers in the afternoon shift. The patient-reported indicators found that the PHCC provided care was of overall high or acceptable quality (81%), that the provided services were “highly needed” by the community (81%, n = 461), and that access to specialised services increased from 45 to 92% from baseline. More than 90% of patients also reported being satisfied with the behaviour of the staff and confident in their knowledge and skills. The report did not detail the number of key informant interviews (including staff) or the total number of respondents. Other documents provided anecdotal evidence of improved access [54]. Three cross-sectional studies provided insights into intermediate health outcomes, focusing on treatment adherence levels. They reported varying levels, ranging from 5 to 70% self-reported adherence [40, 55]. Adherence was significantly lower for hospital patients compared to camp PHCCs in one study and associated with perceived, rather than diagnosed, NCDs in another report [40, 56].

#### Intermediate outcomes

Across the dimensions of the **quality domain**, few dimensions were addressed, often with little detail provided. A common theme was **integration and continuity**, such as referral pathways or follow-up processes. They were mainly covered by commentaries or under recommendations, while no document provided insights into their consistency or success. References to the existence of referral pathways for complicated or specialised cases were made in some settings [39, 45, 50, 52, 53]. One report detailed that most patients were referred to other non-governmental organizations (NGOs; 71%) rather than governmental facilities (29%) [39]. Two documents noted referral gaps, particularly for patients with NCD complications in the KRI and because of the interruption caused by the need for special referral documents in Al-Anbar in 2018 [40, 53]. Follow-up processes equally lacked detailed descriptions. Basic follow-up was reported to be part of MSF’s mobile unit model and generally also available at public hospitals for camp-based refugees across the KRI [48, 55]. In another camp-based setting, an imminent governmental camp-closure threatened continuity, as it did not allow for medical files and a 3-month medicines stock to be prepared [47]. Authors agreed that most international actors generally provided NCD services to fill specific gaps, thus working complementarily to the public healthcare system or providing technical support to the Directorate of Health [40, 45, 49, 50, 57].

**Patient experience** (i.e. acceptability in quality dimensions) was focused on the role of preferred branded medicines and mistrust in Iraq’s public health system [52, 55]. Data about the **quantity** of care was limited to pointing to a high workload of healthcare professionals, thus lacking consultation time for patient education [49, 50, 52]. In one setting, a camp-based PHCC reportedly saw 300 patients per day [50]. **Clinical quality** was only described by one document, mentioning the WHO and health cluster partners’ support to the Directorate of Health for training health professionals [50]. One document called for the training of primary care physicians to care for uncomplicated NCDs [40]. In the **responsiveness** dimension, authors commented on the issue of varying opening times, the limited space in mobile clinics as well as the potential of cash-based assistance for increasing engagement [45, 46, 49].

**Access and coverage** was the most frequently and in-depth discussed domain in this review, mostly centring around accessibility, availability and affordability. The **accessibility** of NCD care (self-reported as having no difficulty in accessing services or medicines), according to population or household access surveys of IDPs, refugees and NGO beneficiaries, varied widely from < 10 to > 90% in some settings [52, 55–58]. Studies used diverse access measures, including having seen a health professional in the last 3 months [40], absence of self-reported access barriers or difficulties [43, 52, 55, 56, 58], or unspecified variables [57]. Refugees residing in camps generally reported good access rates [55, 56]. Reduced access rates were attributed to the greater distance to the nearest city, thus requiring transport, as well as overcrowding and scale of operations, such as generally fewer services being provided in transit camps [41, 42, 45, 52, 54, 55, 57–59]. Access was reportedly differing between the type of facility with the best access reported to camp-based clinics, rather than private or public facilities, for which the worse access rates were noted [43, 44, 55, 56]. While PHCCs generally seemed accessible, in some settings many patients still required transport to reach them [39, 60]. Some documents stratified access rates for specific population groups, noting displaced people living with disabilities or elderly being particularly vulnerable [39, 47, 52, 58]. Access generally dropped during periods of active conflict, influxes of displaced populations and COVID-19 related restrictions [43, 51, 58, 59]. In some settings specific access barriers were reported, such as requirement for referral permits, lack of knowledge about free-of-charge public sector care, or a lack of access to healthy food options [45, 53, 54].

**Availability** and **affordability** focused almost exclusively on **medicines**, with most data from household surveys and some research studies**.** Medicine availability was described as an issue in some settings, and, where data was provided, the reported non-availability ranged widely from < 2 to 45% [44, 45, 50, 53, 55, 57]. While all availability surveys used patient-reported data, their indicators varied. Studies with Syrian refugees used either the presence of medicines at facilities or a persons’ ability to obtain medicines. Two other studies using the same sample reported on medicine availability as a barrier for a patient to take their medicines [40, 41]. They did not find availability differences across facility types [40]. During periods of active conflict, such as in ISIS-occupied Mosul, patient interviews captured a switch from care-seeking in public to private facilities, mostly driven by a more rapid deterioration of medicine availability in the former [43]. In public facilities, insulin prescriptions were reduced to 10-days-a-time. Medicine availability and affordability were intimately connected, as the unavailability of medicines in the public sector frequently forced patients to purchase their medicines elsewhere, often decreasing affordability. Affordability of medicines was a common barrier for camp-based respondents (57–85%) [40, 44, 52]. In one report on displaced persons living in camps, people living with NCDs reported access barriers twice as frequently as the overall sample [55]. Affordability was frequently mentioned as a key barrier (> 80%) in settings where their availability was high [40, 52]. During periods of unaffordability or unavailability of medicines, people reverted to—often unsustainable—coping mechanisms such as rationing their medicines, use of herbal medicines, or selling of personal belongings to fund medicine purchase [41, 43, 46].

Next to medicines, documents addressed **affordability** and **availability** dimensions for healthcare services and equipment. Documents noted that services—as well as medicines—were officially free of charge for displaced persons in all public facilities and some camp-based clinics [47, 55, 59]. One document reported that camp-based PHCCs had the lowest, though still present, associated average cost of care (1.6 USD), compared to the public (4.4 USD) and private (17.3 USD) clinics [41]. Apart from service costs, transport costs were often reported as unaffordable thus creating a major access barrier, particularly for people living in remote camps [41, 45, 52, 54, 58]. Some authors noted availability issues for equipment or affordability issues for laboratory or services [41, 42, 45, 50, 52]. In some locations, such as Sinjar district in 2020, public health facilities were virtually unavailable with 90% of towns and villages facing a lack of health centres [57].

Access concerns around **accommodation** and **acceptability** were infrequent and referred to diverse factors. In relation to medicines availability above, documents observed that self-reported access barriers were frequent even in settings (e.g. camp-based PHCCs) where medicines shortages were rare and services and medicines were generally provided free-of-charge [44, 59]. Alternative explanations may be the perceived unaffordability of care [52], private-sector care seeking [43, 59], lack of knowledge about service availability or them being free-of-charge [45] or preferences of brand medicines [43]. Some studies mentioned a lack of (locally available, suitable or appropriate) human resources, such as a lack of specialists or female care providers in camps [41, 45, 52, 53]. Other mentions of accommodation included physical or language barriers or a mismatch between health counselling content and people’s abilities to follow them [43, 52, 54, 55] and limited opening hours or long waiting times [41, 59]. Three studies captured, though very infrequently, issues of staff rudeness or being refused treatment by the health professional [40, 43, 44]. Only one study reported on the patient perceptions of care, noting that 99% of ‘beneficiaries’ were satisfied or very satisfied with the staff behaviour [39].

#### Patient demands and preferences

For the patient demand and preferences domain (cost and income, knowledge, education, household or cultural characteristics, and distance to service) various factors were touched upon. A cross-sectional survey found that camp households with one or more members with an NCD spent, at the KRI level, 60% more on medical expenses (40,000 IQD/last month) than those without [55]. An influence of wealth on access to care was broadly mentioned by two studies [40, 43]. One document reported that families without income used private sector facilities less frequently (75% of families) compared with those with income (100%) [56]. One source reported that of their sample of 1216 IDP families in various provinces, more than 80% had some income, mainly through gifts, remittances and local charities [60]. In Al-Anbar province, 87% of 355 surveyed IDPs had fixed or temporary income [42]. Lower-income or employment levels were observed for elderly people, women and people living with disabilities [52, 58, 60]. For example, one report noted employment rates of people living with a disability dropping from 94 (n = 979) to 39% because of displacement [52]. The same report highlighted however the role of the family in facilitating access to care [52]. Other themes that were mentioned were frequent overcrowding for displaced populations living in camp shelters [46, 60] and the access to healthcare being worse for those living far from the public health system [41, 54, 55, 57].

#### Socio-cultural, policy and health system context

Many documents focused on the geographical differences in humanitarian responses as well as noting challenging economic circumstances, both for the whole of Iraq and particularly for displaced families. Most IDPs in Iraq are living in the host community (92%) rather than camps-based housing and most are hosted in central Iraq (68%) rather than the KRI (28%), which has seen greater international support due to improved security [56, 60]. Contextual descriptions frequently noted a shift in KRI’s initial open-access policy to more restrictive regulations due to a lack of international support [42, 52, 56, 59, 60]. Effects of economic stressors and reduced public health system financing were reportedly forcing displaced families to move to camps while health professionals were likely to increase their private sector work due to salary delays and reductions [40–42, 44, 60]. Economic impacts seemed to have worsened during the onset of the COVID pandemic [58]. Two documents described changes in October 2020, with an agreement on the Sinjar district being reached and a process of camp closures being initiated [47, 57].

A common theme in this domain was the care-seeking patterns of displaced populations. Most patients sought NCD care in the public healthcare system, both for those residing inside and outside of camps [40–42, 45, 56, 59]. However wide inter-governorate differences were observed [56]. Alongside this trend, there was consistent reporting of families seeking care from private facilities, sometimes as the main access points or where most patients obtained their medicines [40–42, 45]. This may be related both to medicine availability and to mistrust in the public system, particularly by Syrian refugees [52, 55, 56, 60]. Trust in the public system may also depend on the specific NCD, as one study reported different utilisation rates per NCD diagnosis [40]. Private sector care-seeking increased during periods of active conflict, often linked to issues of medicines availability in the public sector [43]. Care seeking was also seen to be influenced by communities’ perceptions and misconceptions of NCDs, such as NCDs being untreatable [40, 41, 44, 46, 56].

Contextual descriptions also highlighted the severe impact of periods of armed conflict in its impacts on living situations, health system capacities and the prevalence of brutality and abuse, particularly against religious minorities [40, 41, 43, 53, 60]. Active conflict further aggravated people’s stress and often led to a down-prioritization of health concerns [40, 42, 43, 53]. Two documents highlighted specific challenges for Yazidi communities, including language barriers and targeted violence [40, 57].

## Discussion

This review assessed the currently available evidence on models of NCD care for displaced populations in Iraq. It adds to global reviews through exploring models of care in Iraq as a single country, allowing broader inclusion criteria and contextualisation [7, 14, 15, 61].

We observed an increase in the volume of publications and documents on NCD models of care for displaced populations in Iraq since 2015, while the depth of evidence varied across settings and framework dimensions. This review showed an increasing number of documents with 77% of those included having been published since 2015. However, the large majority were non-peer-reviewed studies which may be due to the challenges and low priority of conducting research in humanitarian settings [62], as well as limited locally produced research in Iraq, due to lack of support and funding, and shortage of experienced and skilled researchers [63, 64]. Peer-reviewed studies generally used cross-sectional study designs, with no longitudinal studies, which are usually recognised as more robust [62]. Similar gaps in high-quality evidence were identified globally [7]. Contrary to previous global reviews, most included documents in this review addressed NCDs in general rather than specific NCDs, which may be due to the prevalence of reports and press releases. Documents generally addressed only individual framework dimensions, with most focusing on traditional health system building blocks of *facility-based services* and *medicines*. Intermediate outcomes centred around *accessibility*, *availability*, and *affordability*, while the quality of care dimensions and patient demand and preferences were addressed only by around 10% of the papers on average. A few dimensions were not addressed by any document, such as *financing and governance*, *safety*, *knowledge* and *education* dimensions. All documents focused on conflict-affected communities, rather than those impacted by disasters or their synergic impacts. Evidence identified in this review was also skewed towards the KRI (55% of documents) and camp-based populations (45% of documents). This contrasts with most IDPs residing in central Iraq (68%) rather than the KRI in 2016 and outside of camps (92%). This may be due to the centricity of humanitarian response operations in stable areas and with accessible populations [56, 60]. Other groups, such as refugees residing in urban host communities, have often been neglected and have not yet been studied specifically in Iraq [65].

We did not find strong evidence regarding the effectiveness of NCDs models of care for displaced populations in Iraq. The most frequently described model of care was a joint response by camp-based or parallel structures and the formal national healthcare system. Camps typically provided NGO-run PHCC care services, while sometimes including basic referral structures to the public healthcare system. This complementary approach has been called for previously, is appropriate in Iraq’s context and may support sustainability [35]. In this review, we have also observed that integration between these services is non-standardized and often insufficient, which does not mirror the patients’ frequent movement across sectors and facilities. References to diverse implementing actors in this review suggest that there is a range of existing experience of implementing NCD care for displaced populations in Iraq, but little is known about outcomes or effectiveness. To address this, there is a need for routine data collection and implementation research. In other settings, increasing evidence is being generated about the usefulness of approaches including peer-support groups, standardization of protocols, digital cohort monitoring and task-shifting, but we did not identify any evidence of these in this review [30, 35, 48, 66–72]. Evidence on NCD patients in Iraq’s host population noted the usefulness and acceptability of single-dose combinations, health education sessions, and m-Health interventions, whose applicability should also be explored for displaced populations [73–75]. The review identified no reports about NCD prevention activities, despite it being a specific objective in Iraq’s national development plan 2018–2022 and feasible in humanitarian settings and post-conflict countries [35, 76, 77]. However, patient education has not systematically been implemented for refugee populations in the region more broadly [72]. While prevention-focused components may be feasible, the limited consultation times, as well as social and cultural aspects, need to be taken into consideration [49, 52, 78].

Access to NCD care varied widely within Iraq and the type of access barriers were highly contextual. Access was the main theme in the included documents, mostly centred around availability, accessibility and affordability with fewer documents addressing acceptability or accommodation. Access was a key issue across most settings that were described. However, in this review, vastly different access rates were observed across governorates and time, similar to what has been described by actors implementing NCD care in the Syria crisis [79]. Limitations to access had varied reasons such as insecurity in most of Iraq’s districts in 2010 [51], due to non-functioning facilities and lack of staff in Sinjar in 2020 [57], an influx of refugees or returnees [58, 59], distance to the nearest city [52, 55], or because of COVID-19 lockdowns in 2020 [58]. These results suggest that even within one country the degree of access or reasons for a lack thereof vary widely, requiring contextual knowledge on local circumstances when designing an appropriate model of NCD care. This review thus reiterates earlier calls to take the regionality of crises into account [35, 79, 80]. Specific access dimensions were often centred around access to medicines, which was reportedly worse in public facilities compared to parallel camp-based PHCCs or private facilities. Where medicine availability was a major barrier it was one of the top priorities to improve NCD care, as has been observed in models of care in sub-Saharan Africa and a case study in Mali [30, 81]. This review has also shown the impact of ongoing or recent conflict on access rates, with many people forced to change or limit their health seeking or care. Depending on the context, minorities were at even greater risk within an already vulnerable population. These impacts reiterate the critical role of the stage of a humanitarian crisis in decisions on potential NCD models of care and their feasibility [35].

Patients’ perception and trust in the health system shape care-seeking patterns. Across camp- and host community-based displaced populations, most people sought care from the national healthcare system—mostly from higher-level facilities or private facilities [41–43, 56]. This pattern may be shaped by the health system’s historic centricity around tertiary-level care and its influence on the perceived quality of care [82]. Nonetheless, primary care facilities—mostly focused on camp-based facilities—remained a critical provider for a large proportion of people across settings. They played a critical role in ensuring equitable access to care, with being more accessible and affordable than any other facility type. This role of the primary sector, as well as high patient satisfaction, was observed previously in a national household survey in Iraq [83]. Primary care should be at the centre of the NCD health system response, yet no evidence has emerged in this review on how decentralisation could be achieved, particularly while addressing patients’ trust and their perception of quality [35, 84]. Patients commonly used private sector facilities complementarily or as their main access point. The reasons for private-sector care-seeking varied but centred around the perceived quality of care or lack of choice. A commonly cited reason was the availability of medicines, including specific brands [43, 52, 55]. When not available, such as during periods of armed conflicts where public facilities faced greater shortages, patients were forced to purchase them at private pharmacies [43]. Other authors highlighted the key role of medicines and health workers availability as reasons [85]. However, patients continued to seek private-sector care even where medicines availability in the public sector was improved and despite additional affordability barriers (e.g., transport costs, service fees) [41]. In this review, the mistrust towards Iraq’s public healthcare system was proposed as a potential reason, particularly by Syrian refugees. Similar patterns on preferences and trust were captured for Syrian refugees in Jordan and Lebanon [72]. Iraq’s largely unregulated private sector and its dual-practice system may further play a role [86, 87]. Income levels seemed to have some impact on private-sector care-seeking, while even families without income commonly accessed private care in one setting [56]. This observation suggests that people may take additional barriers into account to access their preferred care. These patterns reiterate the criticality of developing patient-centric models of care and addressing issues of patient trust and perceived quality, for example through continuous quality assessments at primary level care [88].

## Strengths and limitations

The diverse inclusion criteria, including qualitative and quantitative methods and grey literature documents, allowed a greater depth of insights. Following PRISMA guidelines for scoping reviews, we did not conduct a formal quality appraisal and the included documents may thus be of varying quality and rigour. The grey literature was sought from internationally recognised humanitarian organisations providing some level of quality control. The authors attempted to counteract the varying type of publications and depth of provided data by giving detailed descriptions of each study’s characteristics. Due to the heterogeneity of the included publications, no meta-analysis could be conducted. The fact that the inclusion criteria were limited to materials published in English may have led to the exclusion of potentially relevant papers in Arabic, Kurdish, or other languages. Our study team includes a senior author with substantial experience and knowledge of the national setting, who has guided scoping and data interpretation.

## Conclusions

This review aimed to explore models of NCD care for displaced populations in Iraq. This review concluded that (i) there is a lack of evidence on the effectiveness of NCDs models of care for displaced populations in Iraq, (ii) access rates and barriers are highly contextualised and vary across time, location, and the crisis phases, (iii) primary level NCD care is critical for equitable access, while private sector providers’ contributions play a role even during the worst humanitarian crises, (iv) patients’ perception of care should be a core consideration when designing a model of NCD care. To address the identified gaps, we recommend the strengthening of implementation research and evaluation capacities of humanitarian and academic actors in Iraq to harness existing experiences of implementing models of NCD care. Future research may focus on the effectiveness of NCD models of care with a particular focus on those that are patient-centric and address communities’ perception of care, e.g. patient education, peer-support approaches, or treatment simplifications. Such efforts could build on the applied model of care framework which has proven a useful analysis and comparison tool in this review.

## Data Availability

All data generated or analysed during this study are included in this published article.

## ANNEX A: Searched grey literature platforms and humanitarian actor’s websites

ACAPS, ALNAP, Care International, European Civil Protection and Humanitarian Aid Operations, Handicap International, HelpAge International, Humanitarian Practice Network, Humanitarian Response, International Committee of the Red Cross, International Medical Corps, International Rescue Committee, Médecins Sans Frontières, Prevention Web, Relief Web, United Nations Development Programme, United Nations Children's Fund, United Nations High Commissioner for Refugees, United Nations Office for the Coordination of Humanitarian Affairs, World Health Organization.

## ANNEX B: Sample search—Medline

1. exp Chronic Disease/
2. exp Cardiovascular Diseases/ or exp Cerebrovascular Disorders/
3. exp Hypertension/ or exp Blood Pressure/
4. exp Diabetes Mellitus/ or exp Blood Glucose/
5. exp Obesity/ or exp Hypercholesterolemia/ or exp Cholesterol/
6. (hypertens* or "blood pressure*").mp.
7. ("noncommunicable disease$" or "non-communicable disease$" or ((chronic$ or "long term") adj1 (disease$ or condition$ or illness$)) or NCD?).mp.
8. (diabetes or diabetic? or hyperglyc?emia or "blood sugar" or "blood glucose").mp.
9. ("heart disease$" or cardiomyopathy or stroke$ or "cardiovascular disease*" or cerebrovascular or "heart failure$" or "myocardial infarct*" or angina? or cardiac or aneurysm* or arrhythmia* or "circulatory disorder*" or (Pericarditis or Myocarditis or Endocarditis or Pancarditis or carditis) or isch?em$).mp.
10. (overweight or smoking or ("physical inactivity" or "physical activity") or "unhealthy diets" or "tobacco use" or "harmful use of alcohol" or ("use" adj2 alcohol) or "body mass index" or (obesity or obese) or cholesterol or hypercholesterol?emia).mp.
11. 1 or 2 or 3 or 4 or 5 or 6 or 7 or 8 or 9 or 10
12. exp "health care facilities, manpower, and services"/ or exp "Delivery of Health Care"/ or exp Health Care Sector/ or exp Public Health/ or exp health services administration/ or exp health planning/ or exp health services research/ or exp "outcome assessment (health care)"/ or exp community-based participatory research/
13. (evaluation$ or evaluate or impact? or outcome$ or output$ or efficac* or effective$4 or feasibility).mp.
14. exp Mortality/ or exp morbidity/ or exp prevalence/ or exp incidence/
15. exp economics/ or exp "health care quality, access, and evaluation"/
16. (((access* or quality) adj3 (medicin$2 or care or treatment? or prevention? or healthcare or service?)) or affordability or appropriateness or availability or acceptability or cost? or "out of pocket" or ((care or healthcare or "health care") adj2 (utilization or seeking))).mp.
17. ("health need?" or (morbid* or prevalen* or inciden* or mortalit$3 or death?) or burden or disabilit$3).mp.
18. ((package$ adj2 care) or intervention$ or program$3 or provision or consultation? or management or service$ or preventi* or ((primary or secondary or tertiary) adj2 ("health care" or healthcare or "medical care" or care)) or (treat$3 or treatment$) or screening? or ((medication? or drug? or barrier? or therapy or treatment) adj3 adherence) or "self-care" or (care adj2 standard?) or ("patient-cent*" or "integrated health care") or "public health" or ((service? or care or "health care" or healthcare) adj2 delivery)).mp.
19. 12 or 13 or 14 or 15 or 16 or 17 or 18
20. exp "Refugees"/
21. exp "Warfare and Armed Conflicts"/ or exp Starvation/ or exp Tsunamis/ or exp earthquakes/ or exp climatic processes/ or exp Avalanches/ or exp Landslides/ or exp Disasters/ or exp Hunger/
22. exp Emergency Medicine/ or exp Emergency Medical Services/ or exp Disaster Medicine/ or exp Medical Missions/
23. ("conflict-affected" or "conflict-related" or drought$ or ("disaster-affected" or "disaster-related") or (disaster? adj3 (natural or victim? or plan* or relief or response? or recovery or risk or resilience or complex or preparedness or management or health or planning)) or (typhoon$ or hurricane$ or cyclone$) or (avalanche$ or earthquake$ or flood? or flooding? or flooded or landslide$ or tsunami$) or (starvation or famine$) or sanction? or (during adj2 occupation) or ("complex emergency" or "complex emergencies") or (unstable adj2 (condition? or setting? or situation?)) or (warfare or war?) or ((armed or ongoing or zone? or area? or region$ or part$ or active or internal or situation$) adj3 conflict$)).mp.
24. ((Relief adj2 (work* or aid)) or humanitarian*).mp.
25. ((displace$ adj2 (force$ or population? or human or intern$ or person* or people)) or IDP? or refugee$ or (evacuee or evacuated) or (asylum adj1 seek*)).mp.
26. 20 or 21 or 22 or 23 or 24 or 25
27. exp Iraq/
28. (Kurdistan or Iraq*).mp.
29. 27 or 28
30. veteran*.mp. or exp Veterans/
31. 11 and 19 and 26 and 29
32. 31 not 30

## ANNEX C: Table of the included publications per conceptual framework dimension

**Table:**

	Input domain						
Citation	Community-based services	Facility-based services	Medicines	Technology and equipment	Information	Health workforce	Financing and governance
Baxter et al. [43]		♦					
Boulle et al. [45]		♦		♦	♦		
Bruaene et al. [47]		♦					
Cetorelli et al. [40]		♦	♦			♦	
Cetorelli et al. [41]	♦	♦					
Jadoo et al. [42]		♦					
IRC [48]						♦	
Kiani [49]	♦	♦					
Lafta et al. [44]							
REACH [51]			♦				
REACH [52]		♦					
REACH [53]			♦				
Sa'Da et al. [55]							
Shamsi [56]		♦	♦		♦		
UNHCR [54]		♦	♦				
WHO [59]		♦	♦				
ACAPS [46]							
ICRC [57]							
MSF [58]		♦		♦			
Oxfam et al. [50]							
CARE [39]		♦	♦	♦		♦	
WHO [60]		♦	♦	♦			

	Intermediate outcomes					
Citation	Quantity	Clinical quality	Patient experience	Safety	Responsiveness	Integration and Continuity
Baxter et al. [43]						
Boulle et al. [45]						♦
Bruaene et al. [47]						
Cetorelli et al. [40]		♦				♦
Cetorelli et al. [41]						
Jadoo et al. [42]						
IRC [48]						♦
Kiani [49]	♦		♦		♦	♦
Lafta et al. [44]						
REACH [51]			♦			♦
REACH [52]						
REACH [53]						
Sa'Da et al. [55]						
Shamsi [56]					♦	
UNHCR [54]			♦		♦	♦
WHO [59]	♦		♦			♦
ACAPS [46]						
ICRC [57]						
MSF [58]						♦
Oxfam et al. [50]						
CARE [39]			♦		♦	♦
WHO [60]	♦	♦				♦

	Intermediate outcomes				
Citation	Availability	Affordability	Accessibility	Accommodation	Acceptability
Baxter et al. [43]	♦	♦	♦		♦
Boulle et al. [45]					
Bruaene et al. [47]			♦		
Cetorelli et al. [40]	♦	♦	♦		♦
Cetorelli et al. [41]	♦	♦	♦	♦	
Jadoo et al. [42]	♦		♦		
IRC [48]	♦		♦	♦	
Kiani [49]	♦	♦	♦	♦	
Lafta et al. [44]			♦		
REACH [51]	♦	♦	♦	♦	
REACH [52]	♦	♦	♦	♦	
REACH [53]	♦		♦		
Sa'Da et al. [55]	♦	♦	♦	♦	
Shamsi [56]		♦			
UNHCR [54]	♦	♦	♦	♦	
WHO [59]					
ACAPS [46]	♦		♦		
ICRC [57]	♦	♦	♦	♦	
MSF [58]	♦	♦			
Oxfam et al. [50]		♦	♦		
CARE [39]			♦		♦
WHO [60]	♦				

	Patient demand and preferences						
Citation	Cost & Income	Knowledge	Education	Household/ cultural characteristics	Distance to service	Broader policy	Sociocultural context
Baxter et al. [43]	♦						♦
Boulle et al. [45]							
Bruaene et al. [47]							
Cetorelli et al. [40]	♦						♦
Cetorelli et al. [41]					♦		♦
Jadoo et al. [42]	♦						♦
IRC [48]							♦
Kiani [49]	♦			♦			♦
Lafta et al. [44]	♦						♦
REACH [51]	♦				♦		♦
REACH [52]							♦
REACH [53]	♦						♦
Sa'Da et al. [55]							♦
Shamsi [56]				♦			♦
UNHCR [54]							♦
WHO [59]							
ACAPS [46]					♦	♦	♦
ICRC [57]					♦		♦
MSF [58]						♦	
Oxfam et al. [50]				♦		♦	♦
CARE [39]							
WHO [60]							

## Abbreviations

CARE: Cooperative for assistance and relief everywhere
CVD: Cardiovascular diseases
DM: Diabetes mellitus
DoH: Directorate of health
ECHO: European civil protection and humanitarian aid operations
HICs: High-income countries
HTN: Hypertension
ICRC: International committee of the red cross
IDP: Internally displaced persons
IQD: Iraqi dinar
IRC: International rescue committee
ISIS: Islamic state of Iraq and the Levant
KRI: Kurdistan region of Iraq
LMICs: Low- and middle-income countries
MSF: Médecins sans frontières
NCDs: Non-communicable diseases
NGO: Non-governmental organization
PHCC: Primary health care centre
PRISMA: Preferred reporting items for systematic reviews and meta-analyses
UNHCR: United nations high commissioner for refugees
WHO: World health organization
